# Survey Study to Identify the Maximum Acceptable Numbers of 2 mm and 3 mm Mini-Tablets for Short-, Middle-, and Long-Term Treatments in Acutely and Chronically Sick Children of Different Age Groups Below 18 Years

**DOI:** 10.3390/pharmaceutics17070834

**Published:** 2025-06-26

**Authors:** Manfred Wargenau, Eva Mutzke, Lucas-Sebastian Spitzhorn, Sibylle Reidemeister, Ingrid Klingmann, Viviane Klingmann

**Affiliations:** 1 M.A.R.C.O. GmbH & Co. KG, Institute for Clinical Research and Statistics, 40211 Duesseldorf, Germany; manfred.wargenau@marco-institut.de (M.W.); lucas.spitzhorn@marco-institut.de (L.-S.S.); 2 Department of General Pediatrics, Neonatology and Pediatric Cardiology, Medical Faculty and University Hospital Duesseldorf, Heinrich Heine University, 40225 Duesseldorf, Germany; evasophie.mutzke@med.uni-duesseldorf.de; 3 Novartis Pharma AG, Global Drug Development/Technical Research & Development, Novartis Campus, 4056 Basel, Switzerland; sibylle.reidemeister@novartis.com; 4 Pharmaplex bv, Avenue Saint-Hubert 51, 1970 Wezembeek-Oppem, Belgium; iklingmann@pharmaplex.be

**Keywords:** mini-tablets, pediatric formulation, medicine dosage form, medicinal product administration

## Abstract

**Background/Aims**: This preference study aimed to identify the maximum number of mini-tablets of two different sizes that children and adolescents would be willing to take, and parents, nurses, and pediatricians would be willing to administer to children and adolescents. **Methods**: A total of 336 participants (24 per cohort) were stratified into 14 cohorts: Acutely and chronically sick children/adolescents aged 6–<12 years (y) and 12–<18 y, parents of acutely and chronically sick children/adolescents aged 0–<2 y, 2–<6 y, 6–<12 y, and 12–<18 y, nurses, and pediatricians. After participants were shown seven different numbers of 2 and 3 mm mini-tablets, they were asked to assess the maximum number acceptable for short-, middle-, and long-term treatment using cohort-specific questionnaires. **Results**: Within all groups and treatment durations, the specified maximum accepted number of 2 mm mini-tablets was higher than the number of 3 mm mini-tablets. The maximum number of mini-tablets acceptable to the participants increased with increasing age of the children and ranged from 40–500 (30–400) for the 2 mm (3 mm) mini-tablets. With increasing duration of treatment, a lower maximum number of mini-tablets was considered tolerable. Chronically sick children were willing to take higher numbers of mini-tablets than acutely sick children. The maximum accepted numbers of mini-tablets specified by pediatric patients, parents, and healthcare professionals were similar for each age group; however, pediatricians consistently specified slightly higher numbers of mini-tablets than parents and nurses. **Conclusions**: The study demonstrated that even a high number of mini-tablets would be well-accepted by children during middle- or long-term treatment.

## 1. Introduction

Development of a new medicine for pediatric patients not only requires the investigation of its efficacy and safety in the different age groups but also the development of age-appropriate galenic formulations to ensure more precise and reliable administration [1].

Solid oral dosage forms allow for less dosing flexibility than liquid formulations, but have major advantages because they circumvent problems that are associated with the use of liquids, such as higher production costs, potentially toxic excipients, complicated storage conditions [2], taste-masking [3], and inaccurate dosing. A variety of alternative pediatric drug formulations have been developed, such as mini-tablets of different sizes, oblong tablets, orodispersible tablets, orodispersible mini-tablets, and buccal and orodispersible films.

In the clinical setting, the treatment of pediatric patients with medicines is impeded by a shortage of available licensed products in the appropriate formulation. The specific dosing requirements depend on the age and physical development stage of the child, but unavailability of the required strength of the formulation, the child’s inability to ingest standard-size solid dosage forms, and the taste of oral medicines often result in selection of other formulations such as liquid or suppository [4] or the manipulation of the available drug before administration [5]. Due to the fact that the use of off-label or even unauthorized medication is more frequent in the pediatric population, children have a higher risk of facing adverse drug reactions resulting from this practice [6].

Few scientifically sound data on the suitability of different formulations in children of different age groups are available, and there are concerns and uncertainties amongst clinicians about the age at which young children are able to safely swallow solid oral medications such as conventional tablets and capsules. Khan et al. [7] published a review of the current stage of pediatric formulation development, stating: “Considering the extensive variability between the paediatric populations, a systematic approach to paediatric formulation development should be employed to ensure safe and effective treatment, where paediatric specific drug delivery systems are available which contain non-toxic excipients, are palatable, grant minimal dosage and frequency, are applicable to all ages and exhibit easy and reliable administration.” Also, there is a huge variability in the methodologies that are used to assess the acceptability of pediatric formulations, indicating that there is a need for a more standardized evaluation method [8].

Mini-tablets have advantages over liquid formulations as they do not need reconstitution, offer advantageous dosing accuracy, usually have favorable storage conditions, shelf-life, no in-use period, and allow better drug stability [3]. For evaluating the suitability of mini-tablets for the pediatric population, the working group of the Pediatric Clinic of the University Hospital Duesseldorf performed eight clinical studies [9,10,11,12,13,14,15,16] in over two thousand patients aged 0–18 years (y) to investigate the acceptability of different mini-tablet formulations, administered in single and up to 400 mini-tablets with a reliable, validated, investigator-performed method to assess swallowability and palatability. Based on the results of this research, the revised version of the European Medicines Agency (EMA) Guideline from 2014 [17] no longer provides any age recommendation for solid oral dosage forms. In most studies, the mini-tablets proved to be more acceptable than syrup. Also, oblong tablets (2.5 × 6 mm) have already been investigated and showed very good acceptability and swallowability [13].

Other research teams have applied questionnaire methods on the preferences of oral formulations in limited populations [8]. To evaluate the suitability of this assessment method, our team performed a questionnaire study to investigate whether the swallowability and palatability of different oral dosage forms in children and adolescents differ from the preferences expressed by parents, nurses, pediatricians, and older children (6–<18 y) when they are offered various formulation options [18]. This patient- and user-centric study showed results similar to the clinical trials in which the different formulations were administered: mini-tablets were favored by children, adolescents preferred oblong tablets compared to multiple mini-tablets, whereas round tablets and syrup were less preferred. Little is known about the maximum acceptable quantity of different sizes of mini-tablets for children and adolescents in short-, medium-, and long-term treatments.

The present questionnaire study was performed to determine the maximum acceptable number of mini-tablets of different sizes for three different treatment regimens from the perspectives of children and adolescents aged 6–<18 y as well as parents, nurses, and pediatricians for all age groups from newborn to aged <18 y. As the experience with oral drugs as well as the duration of treatment could have an effect on the subjects’ preferences, we divided the cohorts into acutely and chronically sick patients and their parents.

## 2. Materials and Methods

### 2.1. Objectives

The primary objectives of this study were to identify the maximum number of 2 mm as well as 3 mm mini-tablets that acutely and chronically sick children aged 6–<12 y and adolescents aged 12–<18 y would be willing to take. The secondary objectives were to identify the maximum number of 2 mm as well as 3 mm mini-tablets that parents of acutely or chronically sick pediatric patients aged 0–<2 y, 2–<6 y, 6–<12 y, and 12–<18 y would be willing to administer to their child and to identify the maximum number of 2 mm as well as 3 mm mini-tablets that nurses and pediatricians would be willing to administer to pediatric patients aged 0–<2 y, 2–<6 y, 6–<12 y, and 12–<18 y.

### 2.2. Study Design and Cohort Definitions

This was a survey study using adapted question sets for 14 cohorts, i.e., acutely and chronically sick children aged 6–<12 y, acutely and chronically sick adolescents aged 12–<18 y, parents of acutely and chronically sick children/adolescents in four age groups (0–<2 y, 2–<6 y, 6–<12 y, 12–<18 y), nurses of acutely and chronically sick children/adolescents in four age groups (0–<2 y, 2–<6 y, 6–<12 y, 12–<18 y), and pediatricians of acutely and chronically sick children/adolescents in four age groups (0–<2 y, 2–<6 y, 6–<12 y, 12–<18 y) (see Table 1). Nurses and pediatricians, thereby, were asked about all age groups at the same time. Each participant had to answer all questions.

The study received a favorable opinion from the Ethics Committee of the Medical Faculty of the Heinrich Heine University Duesseldorf (No. 2023-2616, 11.10.2023), was registered in the German Clinical Trial Register (No. DRKS00032860), and performed in accordance with the ethical principles which have their origin in the Declaration of Helsinki and which are consistent with Good Clinical Practice (ICH-GCP(R2)) and applicable regulatory requirements.

### 2.3. Study Population

All participants in this study were recruited from the Department of General Pediatrics, Neonatology, and Pediatric Cardiology of the University Hospital Duesseldorf, Germany. The children and adolescents were pediatric patients registered either as inpatients or outpatients at the University Hospital. 50% of the children and adolescents (pediatric patients) in each age group were acutely sick, and 50% were chronically sick patients. Also, in each group of acutely and chronically sick pediatric patients, 50% were male and 50% were female patients. Half of the parents in each age group were parents of chronically sick children/adolescents, whereas the other half were parents of acutely sick children/adolescents. Nurses and pediatricians were questioned regarding both acutely and chronically sick pediatric patients in all four age groups. For the study, a total of 336 evaluable paper-based case report forms (CRFs) (24 per cohort) were included. Very few of the participants in the study had had any experience in either taking or administering mini-tablets.

### 2.4. Inclusion Criteria

The following inclusion criteria were applied:

(1) Pediatric patients aged 6–<18 y, of whom 50% had to be male, parents of pediatric patients aged 0–<18 y, health care professionals (nurses and pediatricians) taking care of pediatric patients aged 0–<18 y;

(2) Pediatric patients aged 6–<18 y had to be either acutely or chronically sick;

(3) Parents and healthcare professionals taking care of acutely or chronically sick children/adolescents: Following the definition by Mokkink et al. [19], a participant is considered to be chronically sick if (i) the underlying disease or condition lasts 3 months or more or has occurred 3 times or more during the past year and will probably reoccur, (ii) affects a child’s normal activities and requires frequent hospitalizations, home healthcare, and/or extensive medical care, and (iii) the underlying disease or condition is not (yet) curable or is highly resistant to treatment;

(4) Participants had to be capable of understanding the survey procedures and questions and indicated their consent verbally.

### 2.5. Exclusion Criteria

The following exclusion criteria were applied:

(1) Pediatric patients aged 6–<18 y unable to read and understand the questionnaire on their own (e.g., due to cerebral palsy);

(2) Pediatric patients who were not fully awake yet, e.g., in the post-operative period;

(3) Pediatric patients who were not fully oriented, regardless of the reason.

### 2.6. Study Procedure

After giving their consent, participants were allocated a unique identification number in chronological order of enrolment. All information and instructions were given by the investigator in a standardized manner using age-appropriate language. The participants were seated in a quiet, distraction-free area. The investigator completed the paper-based CRF documenting the participants’ answers to each question. After completion of the basic information, the investigator presented the different numbers of uncoated mini-tablets of both sizes (2 mm and 3 mm) relevant for the respective participant cohort simultaneously in transparent glass containers, and the participant was encouraged to answer the questions. Pediatric patients, parents of pediatric patients, nurses, and pediatricians were shown seven different numbers of mini-tablets with a diameter of 2 mm and 3 mm. The numbers varied according to the respective age group (Table 2) and were selected based on experiences from previous studies. The aim of this procedure was to enable the participants to estimate the maximum number of mini-tablets they would be willing to take or administer. For all questions, the chosen maximum number of mini-tablets was unlimited, any number could be specified, so not necessarily one of the presented numbers had to be noted. Questions addressed to the investigator for further clarification could be asked at any time.

### 2.7. Description of Questionnaires

Three types of questionnaires were used to evaluate the preferences for the different mini-tablet numbers/sizes. The questionnaires were designed by the M.A.R.C.O. GmbH & Co. KG (Duesseldorf, Germany) with support of the whole study team.

Type A questionnaires were used for pediatric patients aged 6–<18 y:

(1) Previous experience with mini-tablets (“Ever taken?”: yes/no);

(2) Age, sex, and health status (acutely/chronically sick);

(3) Maximum number of mini-tablets they would be willing to take as single administration for each tablet size;

(4) Maximum number of mini-tablets they would be willing to take 3 times a day for one week for each tablet size;

(5) Maximum number of mini-tablets they would be willing to take 3 times a day for 12 months for each tablet size.

Type B questionnaires were used for parents of pediatric patients aged 0–<18 y:

(1) Previous experience with mini-tablets (“Ever given to the child?”: yes/no);

(2) Age, sex, and health status of their child (acutely/chronically sick);

(3) Maximum number of mini-tablets they would be willing to administer to their child/adolescent as single administration for each tablet size;

(4) Maximum number of mini-tablets they would be willing to administer to their child/adolescent 3 times a day for one week for each tablet size;

(5) Maximum number of mini-tablets they would be willing to administer to their child/adolescent 3 times a day for 12 months for each tablet size.

Type C questionnaires were used for healthcare professionals:

(1) Years of experience in pediatric area categorized as 0–<5 y, 5–<10 y, and ≥10 y;

(2) Experience with mini-tablets for each of the 4 age groups (“Ever administered?”: yes/no);

(3) Maximum number of mini-tablets they would be willing to administer to a pediatric patient as single administration for each tablet size, separately for each subgroup (i.e., acutely and chronically sick) within each age group;

(4) Maximum number of mini-tablets they would be willing to administer to a pediatric patient 3 times a day for one week for each tablet size, separately for each subgroup (i.e., acutely sick and chronically sick) within each age group;

(5) Maximum number of mini-tablets they would be willing to administer to a pediatric patient 3 times a day for 12 months for each tablet size, separately for each subgroup (i.e., acutely sick and chronically sick) within each age group.

### 2.8. Statistical Analysis

The inclusion of 24 pediatric patients per age and health status group, and 24 parents of pediatric patients per children’s age and health status group, as well as 24 nurses and 24 pediatricians, was considered appropriate for descriptive purposes. For the purpose of sample size estimation, a normal distribution was assumed for the maximum number. However, it was expected that the accepted maximum number of mini-tablets, as well as the variability, may increase with age group. A hypothetical scenario was assumed, in which there is an effect size of 0.667 in all age groups, which was calculated as the quotient of the mean differences between the two tablet sizes and the standard deviation. With α = 5% (two-sided) and power = 80%, N = 20 per cohort would have been required. Since the analysis was performed non-parametrically, a sample size of 24 in each cohort was considered appropriate, taking the loss in efficiency into account (compared to parametric analysis).

In total, 336 questionnaires (24 per cohort) were answered by participants and included in the analyses using descriptive statistical methods. All participants were included in the statistical analysis set. Exploratory and descriptive statistical methods were applied. Summary statistics for maximum numbers of mini-tablets (N, arithmetic mean, SD, minimum, Hodges–Lehmann estimator for the median (HL median), maximum) are presented by cohort, subgroups, and tablet size. Summary statistics were calculated for the difference between tablet sizes with regard to the maximum number of mini-tablets by cohort and subgroups. In addition, rank-based repeated analysis of variance was applied for the primary endpoint, including effect for tablet size, treatment regimen, and health condition. *p*-values are to be interpreted in the exploratory sense.

## 3. Results

### 3.1. Pediatric Patients 6–<18 y

**Figure 1:**
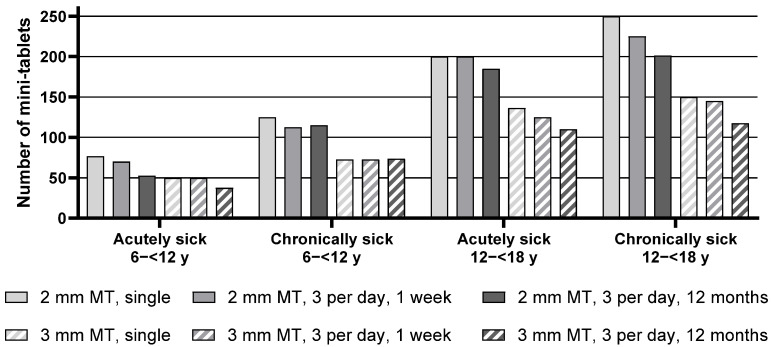
Median of maximum number of mini-tablets in Cohorts 1 to 4. MT = mini-tablets, *n* = 24 for all groups, y = year.

Descriptive statistics for the maximum number of mini-tablets acutely sick and chronically sick pediatric patients from the age groups 6–<12 y as well as 12–<18 y would be willing to take are presented in Table S1, whereas the median of the maximum number of mini-tablets is presented in Figure 1 below.

When comparing the maximum number (median) of mini-tablets of both sizes, the number of 2 mm mini-tablets was found to be significantly higher in all groups for each regimen. For example, acutely sick children aged 6–<12 y noted 76.25 mini-tablets with a size of 2 mm as the maximum number they would be willing to take for a single administration, whereas it was only 50.0 mini-tablets with a size of 3 mm.

Analysis of the effect of the treatment regimen on the maximum number of mini-tablets revealed an overall significant difference between the three treatment regimens, indicating, i.e., with increasing time of the treatment period, a lower maximum number of acceptable mini-tablets was noted. In particular, for chronically sick adolescents aged 12–<18 y, the maximum number of 2 mm mini-tablets they would be willing to take decreased from 250 for a single administration to 225 for an intake 3 times a day for one week, and further to 201.25 for an intake 3 times a day for 12 months. This effect was descriptively observed within each group, except for the chronically sick children 6–<12 y.

Overall, a trend was apparent that chronically sick children were willing to take slightly higher numbers of mini-tablets than acutely sick children. This effect appeared most pronounced for children aged 6–<12 y. Acutely sick children from this group noted 76.25 mini-tablets (2 mm) as maximum for a single administration, whereas chronically sick children noted 125 mini-tablets. However, this trend was not statistically significant for adolescents aged 12–<18 y.

Overall, adolescents aged 12–<18 y were willing to take a higher number of mini-tablets compared to children aged 6–<12 y (see Table S1, Figure 1, and Table 3).

### 3.2. Parents and Healthcare Professionals of Pediatric Patients 0–<2 y

**Figure 2:**
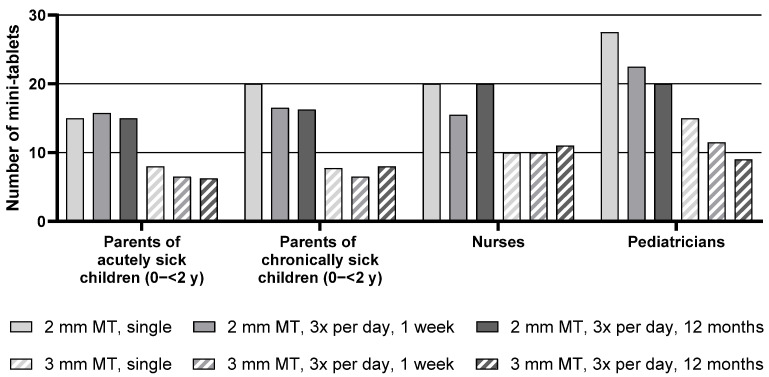
Median of maximum number of mini-tablets for children aged 0–<2 y. MT = mini-tablets, *n* = 24 for all groups, y = year.

Descriptive statistics for the maximum number of mini-tablets parents and healthcare professionals would be willing to administer to children aged 0–<2 y are presented in Table S2, whereas the median of the maximum number of mini-tablets is presented in Figure 2 below.

For the group of children aged 0–<2 y, the median maximum number of 2 mm mini-tablets that parents or healthcare professionals would be willing to administer was higher than the number of 3 mm mini-tablets for all groups and treatment durations. For example, parents of acutely sick children noted a maximum of 15 mini-tablets with a size of 2 mm for a single administration compared to only 8 mini-tablets with a size of 3 mm.

Overall, there was an effect of the treatment regimen on the maximum number of mini-tablets: with increasing treatment duration, a lower number of mini-tablets was considered appropriate. When looking at each group separately, this effect was apparent for the parents of chronically sick children and most pronounced for the pediatricians. For this group, a decrease in the maximum number of 2 mm mini-tablets from 27.5 for a single administration down to 22.5 for an administration period of 3 times a day for 1 week, and further down to 20 for an administration period of 3 times a day for 12 months was observed. Conversely, the numbers noted by parents of acutely sick children and nurses were quite constant over the three treatment durations.

With regard to the health status of the child, no differences were observed between parents of acutely sick and of chronically sick children.

Over all groups, pediatricians noted the highest tablet numbers (see Table S2, Figure 2, and Table 4).

### 3.3. Parents and Healthcare Professionals of Pediatric Patients Aged 2–<6 y

**Figure 3:**
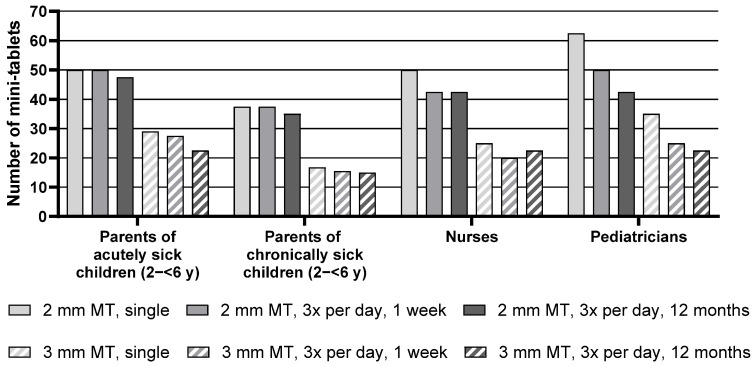
Median maximum number of mini-tablets for children aged 2–<6 y. MT = mini-tablets, *n* = 24 for all groups, y = year.

Descriptive statistics for the maximum number of mini-tablets parents and healthcare professionals would be willing to administer to children aged 2–<6 y are presented in Table S2, whereas Figure 3 below presents the median of the maximum number of mini-tablets.

Regarding the group of pediatric patients aged 2–<6 y, the median maximum number of 2 mm mini-tablets that parents or healthcare professionals would be willing to administer was higher than the number of 3 mm mini-tablets for all groups and treatment durations. Nurses, for example, noted 50 mini-tablets with a size of 2 mm but only 25 larger-sized mini-tablets for a single administration.

Overall, there was an effect of the treatment regimen, i.e., a lower number of mini-tablets was considered appropriate with increasing treatment duration. When looking at each group separately, this effect was observed for the nurses and was most pronounced for the pediatricians. Pediatricians noted a maximum of 62.5 mini-tablets with a size of 2 mm they would be willing to administer for a single administration. For a treatment period of 3 times a day for one week, this maximum number decreased to 50, and even further to 42.5 for an administration period of 3 times a day for 12 months. For the parents of acutely and chronically sick children, this effect was not indicated.

Parents of chronically sick children noted markedly lower maximum numbers than parents of acutely sick children. In particular, for a single administration of 2 mm-sized mini-tablets, parents of chronically sick children noted only 37.5, whereas parents of acutely sick children noted 50 mini-tablets. However, this difference was not reflected by the statistical test result due to the lack of power of the underlying inter-subject comparison (see Table S2, Figure 3, and Table 4).

### 3.4. Parents and Healthcare Professionals of Pediatric Patients Aged 6–<12 y

**Figure 4:**
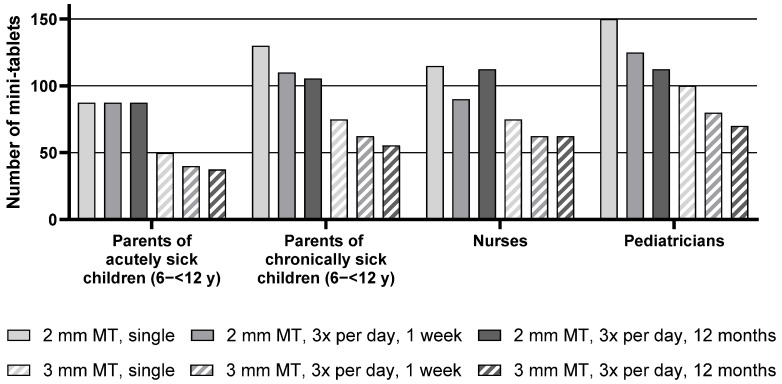
Median maximum number of mini-tablets for children aged 6–<12 y. MT = mini-tablets, *n* = 24 for all groups, y = year.

Descriptive statistics for the maximum number of mini-tablets parents and healthcare professionals would be willing to administer to children aged 6–<12 y are presented in Table S2, whereas the median of the maximum number of mini-tablets is presented in Figure 4 below.

For the group of pediatric patients aged 6–<12 y, the median maximum number of 2 mm mini-tablets that parents or healthcare professionals would be willing to administer was higher than the number of 3 mm mini-tablets for all groups and treatment durations. As an example, parents of chronically sick children noted a maximum of 130 mini-tablets of a size of 2 mm but only 75 larger-sized mini-tablets for a single administration.

The number of acceptable mini-tablets decreased with an increase in duration of treatment. When looking at each group separately, this effect was apparent for the nurses and most pronounced for the parents of chronically sick children and pediatricians. Pediatricians noted 150 mini-tablets (size 2 mm) as a maximum for a single administration in this age group. For a treatment period of 3 times a day for one week, this number decreased to 125 and further to 112.5 for a year-long treatment. For the parents of acutely sick children, this effect was not observed as they noted very similar maximum numbers of mini-tablets for all treatment durations.

Parents of acutely sick children noted lower maximum numbers than parents of chronically sick children. In particular, for a treatment period of 3 times a day for one week with the 2 mm mini-tablets, parents of chronically sick children noted 110 mini-tablets as a maximum, whereas parents of acutely sick children noted only 87.5. However, this difference was not reflected by the statistical test result due to the lack of power of the underlying inter-subject comparison.

It can be seen that pediatricians noted slightly higher numbers than the other groups (see Table S2, Figure 4, and Table 4).

### 3.5. Parents and Healthcare Professionals of Pediatric Patients Aged 12–<18 y

**Figure 5:**
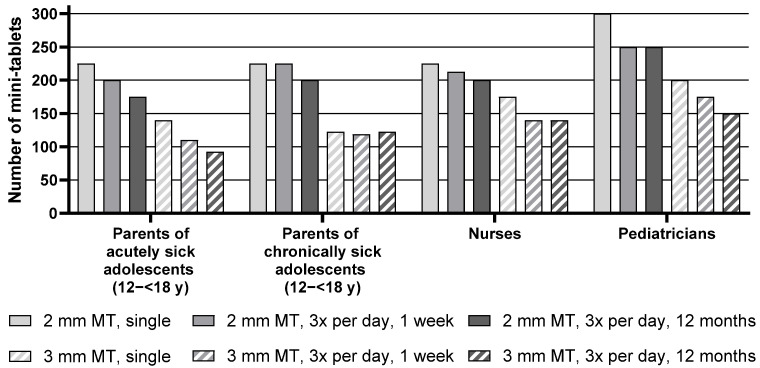
Median of maximum number of mini-tablets for adolescents aged 12–<18 y. MT = mini-tablets, *n* = 24 for all groups, y = year.

Descriptive statistics for the maximum number of mini-tablets parents and healthcare professionals would be willing to administer to children aged 12–<18 y are presented in Table S2, whereas Figure 5 below shows the median of the maximum number of mini-tablets.

For the age group of 12–<18 y, the median maximum number of 2 mm mini-tablets that parents or healthcare professionals would be willing to administer was higher than the number of 3 mm mini-tablets for all corresponding treatment durations for all groups. For example, pediatricians noted a maximum of 300 smaller-sized mini-tablets, whereas they only noted 200 larger-sized mini-tablets for a single administration.

Overall, there was an effect of the treatment regimen, i.e., a lower number of mini-tablets was considered appropriate with increasing treatment duration. When looking at each group separately, this effect was very pronounced for the parents of acutely sick adolescents, nurses, and pediatricians. In particular, parents of acutely sick adolescents noted 225 mini-tablets (2 mm) for a single administration but only 200 for a treatment period of 3 times a day for one week. For an administration period of 3 times a day for 12 months, the number further decreased to 175. For the parents of chronically sick adolescents, this effect was not observed as they noted very similar maximum numbers of mini-tablets for the three regimens.

No influence of the health conditions of the adolescent was seen with regard to the maximum number of mini-tablets as judged by the parents, as the numbers noted by parents of acutely sick adolescents were quite similar to those of parents of chronically sick adolescents (see Table S2, Figure 5, and Table 4).

## 4. Discussion

The aim of this preference study was to identify the maximum number of mini-tablets (2 mm and 3 mm) that children and adolescents would be willing to take, and parents, nurses, and pediatricians would be willing to administer to children and adolescents for short-, middle-, and long-term treatments. A total of 336 participants (24 per cohort) were stratified into the 14 cohorts: acutely and chronically sick children/adolescents aged 6–<12 y and 12–<18 y, parents of acutely and chronically sick children/adolescents aged 0–<2 y, 2–<6 y, 6–<12 y, and 12–<18 y, and nurses and pediatricians of pediatric patients in the respective age groups.

It should be noted that very few participants had had experience with the intake or administration of mini-tablets due to the fact that, currently, few pediatric medicines are available as mini-tablets. The number of mini-tablets that were presented to the participants in each group increased with the ascending age of the group. As anticipated, the maximum number of mini-tablets considered appropriate, irrespective of the size of the mini-tablets, increased in all cohorts with increasing age of the children. This was anticipated as anatomical differences in the different age groups are apparent. From 0–18 y, the whole body and in particular the head/mouth system with the oral cavity are growing in size. Therefore, the higher body mass as well as the potential to ingest higher volumes drives the children/adolescents and their caregivers to state higher numbers of mini-tablets with increasing age.

The study revealed that in each of the three treatment scenarios (single administration, 3 times a day for 1 week, and 3 times a day for 12 months), the specified maximum number of 2 mm mini-tablets was higher than that of the 3 mm mini-tablets. This seems to be logical, since a defined number of 2 mm mini-tablets has a lower volume (looks smaller) than the same number of 3 mm mini-tablets. Possibly, the smaller appearance of the 2 mm mini-tablets could be thought to be less of a burden for the patient.

When comparing the maximum number of mini-tablets between self-assessing pediatric patients, parents, and healthcare professionals, it was seen that the numbers were similar for each age group. However, pediatricians always specified slightly higher numbers of mini-tablets compared to parents, nurses, and the self-assessing pediatric patients. The reason for this might be related to the role of a pediatrician. The authors assume that pediatricians have a stronger focus on the treatment itself, whereas nurses and parents additionally consider other (soft or more practical) factors, such as the well-being and comfort of the patients. This differing viewpoint could result in nurses and parents being a bit more cautious when it comes to the maximum number of mini-tablets they would administer to a child, since they want to limit the burden for the child.

Analyzing the effect of the three different treatment regimens on the maximum number of mini-tablets showed an overall significant difference between the three treatment regimens. With increasing duration of the treatment period, a lower maximum number of mini-tablets was deemed to be appropriate. This effect was also descriptively observed within each group, with the exception of the chronically sick children aged 6–<12 y. If a disease scenario would make it necessary to administer high numbers of mini-tablets for a middle- or long-term treatment, physicians should not have any concerns regarding patient compliance or treatment burden, since higher numbers of mini-tablets are also likely to be tolerated by the children as well.

In both self-assessing age groups (6–<12 y and 12–<18 y), chronically sick children/adolescents were willing to take higher numbers of mini-tablets than acutely sick children/adolescents, although less pronounced for the 3 mm mini-tablet in the age group 12–<18 y. This might be explained by the patients’ experience with long-term oral medication due to their chronic disease. Parents were also willing to administer more mini-tablets to chronically than to acutely sick children for the age group 6–<12 y, whereas this was not the case for the 12–<18 y age group. Interestingly, parents were willing to administer more mini-tablets to acutely than to chronically sick children for the age group 2–<6 y, whereas this was not the case for the 0–<2 y age group.

Very large ranges for the maximum number of acceptable mini-tablets were observed across all cohorts. Depending on the cohort, the minimal stated number was up to 150-fold smaller than the maximum stated number. These large differences might be explained by the lack of experience in nearly all participants with this kind of formulation.

When comparing the results of this study with previous studies on mini-tablets, it can be seen that, on the one hand, the numbers of mini-tablets in this study are much higher than the numbers of mini-tablets used in our last questionnaire [18] and acceptability study [16], in which up to 70 mini-tablets (2 mm) for the age group 12–<18 y were used and also higher than the 4–5 mini-tablets (2 mm) which were well swallowed by children aged 0.5–2 y as shown by Mitsui et al. [20]. On the other hand, the numbers of mini-tablets in this study are lower than the upper maximum numbers of 100 mini-tablets (2 mm) for the age group 0.5–2 y and 400 mini-tablets (2 mm) for the age group 2–<6 y that were shown to have a high acceptability in one of our other studies [12]. However, in that study, only a single swallowing act of the respective mini-tablets took place, and, therefore, these results offer little information with respect to middle- or long-term treatments requiring multiple dosing. Therefore, it would be interesting to verify the outcomes of the present questionnaire study with acceptability studies in the future, where acceptability assessment would be based on swallowability and palatability using the composite endpoint method [21].

To set the present findings into a real-world context, the noted maximum numbers of mini-tablets are compared with the dose recommendation of commercially available mini-tablet products. In 2020, Slenyto^®^ from Neurim Pharmaceuticals (Tel Aviv, Israel) was approved for the treatment of insomnia in pediatric patients 2–18 y [22]. These mini-tablets have a size of 3 mm and are available with 1 mg and 5 mg of melatonin. The recommended maximum dose of 10 mg would correspond to a maximum of 10 mini-tablets with 1 mg [23]. Recently, Aqumeldi^®^ from Proveca Pharma (Dublin, Ireland) was approved for the treatment of heart failure in children from birth to <18 y [22]. These mini-tablets have a size of 2 mm and contain either 0.25 mg or 1 mg of enalapril maleate. As a maximum maintenance dose 0.3 mg/kg is recommended [24]. For example, a 40 kg patient would require 12 mini-tablets with 1 mg or 48 with 0.25 mg. These numbers are clearly within the framework of the results of this study.

A limitation of the study is that the participants were from a single hospital in Germany. Therefore, the results may not be fully applicable to other cultures or medical settings. To address this point, similar studies need to be performed in other countries or regions of the world to exclude any cultural or health system-based bias. Due to the nature of this study, potential influencing factors such as taste (e.g., bitter versus tasteless), mode of administration (e.g., taken with a drink versus soft food), time of application (day or night), or the use of dosing tools were not considered in the survey. These factors may have an impact on the perceived acceptability and feasibility of administering larger numbers of mini-tablets. Therefore, the results of this study could be verified in a follow-up study in which the participants would actually ingest the corresponding numbers of mini-tablets and, therefore, allow inclusion of the above-mentioned variabilities in the judgment. As assessed by the questionnaires, almost none of the participants had any experience with the intake or administration of mini-tablets. This was expected, since mini-tablets are rarely used in standard therapies. However, it would be very interesting to repeat this study (at least partially) in a cohort with experience in the use of mini-tablets to better contextualize the present results.

## 5. Conclusions

This study showed that the maximum number of mini-tablets considered to be appropriate and tolerable increased with increasing age, and decreased with duration of treatment. Chronically sick children were willing to take higher numbers of mini-tablets than acutely sick children. Therefore, the age of the patient and the duration of the planned treatment, as well as the medical history (the presence of an acute or chronic disease), should be considered when developing future mini-tablet therapies for children and adolescents. Also, the acceptability of larger numbers of mini-tablets should be taken into consideration by developers or physicians for middle- or long-term treatments of children, since these are likely to be well tolerated.

## Figures and Tables

**Table 1:** Definition of cohorts.

Cohort	Description	Subgroup	Mean Age of the Child [Years] ± SD	*n*
1	Children 6–<12 y	Acutely sick *	8.7 ± 1.9	24
2		Chronically sick *	8.9 ± 1.7	24
3	Adolescents 12–<18 y	Acutely sick *	14.7 ± 1.8	24
4		Chronically sick *	14.6 ± 1.9	24
5	Parents of children 0–<2 y	Acutely sick	0.3 ± 0.4	24
6		Chronically sick	0.4 ± 0.5	24
7	Parents of children 2–<6 y	Acutely sick	3.0 ± 1.0	24
8		Chronically sick	3.8 ± 1.3	24
9	Parents of children 6–<12 y	Acutely sick	8.5 ± 2.0	24
10		Chronically sick	8.5 ± 1.6	24
11	Parents of adolescents 12–<18 y	Acutely sick	14.2 ± 1.6	24
12		Chronically sick	14.4 ± 1.8	24
13	Nurses	All	-	24
14	Pediatricians	All	-	24
	Total			336

SD = standard deviation, y = year. * Subgroup consisted of 50% female (*n* = 12) and 50% male (*n* = 12) participants.

**Table 2:** Study formulations (placebo) presented to the participants.

	Age Group			
Dosage Form	0–<2 y	2–<6 y	6–<12 y	12–<18 y
No. of 2 mm and 3 mm MT	1, 3, 10, 20, 30, 40, 50	5, 10, 20, 25, 50, 75, 100	11, 25, 50, 75, 100, 150, 200	35, 70, 100, 150, 200, 250, 300

MT = mini-tablets, No. = number, y = year.

**Table 3:** Test results for comparisons between tablet size, treatment regimens, and health conditions for pediatric patients aged 6–<18 y.

Cohort.	
Comparison: Size (2 mm vs. 3 mm)	
Overall	*p*-Values * < 0.0001 for all age groups and cohorts
Acutely sick children 6–<12 y	
Chronically sick children 6–<12 y	
Acutely sick adolescents 12–<18 y	
Chronically sick children 12–<18 y	
Comparison of Regimens (SD, 1 W, 12 M), *p*-Values *	
Overall	0.0010
Acutely sick children 6–<12 y	0.0543
Chronically sick children 6–<12 y	0.6640
Acutely sick adolescents 12–<18 y	0.0637
Chronically sick children 12–<18 y	0.0206
Comparison: Health condition (Acutely vs. chronically sick), *p*-Values *	
Overall	0.0929
2 mm	0.0557
3 mm	0.1731
Children 6–<12 y	0.0573
Adolescents 12–<18 y	0.3885

SD = single administration, 1 W = 3 times a day for one week, 12 M = 3 times a day for 12 months, N = 24 for all groups, y = year, * Resulting from rank-based repeated analysis of variance. Green color indicates *p* < 0.05, and blue 0.05 < *p* < 0.10.

**Table 4:** Test results for comparisons between tablet size, treatment regimens, and health conditions for each age group concerning assessments by parents and healthcare professionals.

Cohort	Age Groups			
	0–<2 y	2–<6 y	6–<12 y	12–<18 y
Comparison: Size (2 mm vs. 3 mm)				
Overall	*p*-values * < 0.0001 for all age groups and cohorts			
Parents of acutely sick children				
Parents of chronically sick children				
Nurses				
Pediatricians				
Comparison of Regimens (SD, 1 W, 12 M), *p*-Values *				
Overall	0.0017	<0.0001	<0.0001	<0.0001
Parents of acutely sick children	0.1545	0.1545	0.3170	0.0015
Parents of chronically sick children	0.0559	0.2928	0.0154	0.2984
Nurses	0.3613	0.0621	0.0533	0.0058
Pediatricians	0.0020	0.0004	0.0155	0.0002
Comparison: Health condition (Acutely vs. chronically sick), *p*-Values *				
Parents of sick children, overall	0.7830	0.4693	0.1620	0.6196
Parents of sick children, 2 mm	0.5983	0.7056	0.3357	0.5536
Parents of sick children, 3 mm	0.9146	0.2600	0.0668	0.7756

SD = single administration, 1 W = 3 times a day for one week, 12 M = 3 times a day for 12 months, *n* = 24 for all groups, y = year, * Resulting from rank-based repeated analysis-of-variance. Green color indicates *p* < 0.05, and blue 0.05 < *p* < 0.10.

## Data Availability

Individual participant data from this study will be available in an anonymized form upon request. Proposals should be directed to the corresponding author.

## Supplementary Materials

The following supporting information can be downloaded at: https://www.mdpi.com/article/10.3390/pharmaceutics17070834/s1, Descriptive statistics for numbers of mini-tablets for all cohorts (Tables S1 and S2).
