# Supplementary material for: Survey Study to Identify the Maximum Acceptable Numbers of 2 mm and 3 mm Mini-Tablets for Short-, Middle-, and Long-Term Treatments in Acutely and Chronically Sick Children of Different Age Groups Below 18 Years
Source: Pharmaceutics. 2025 Jun 26;17(7):834. doi: 10.3390/pharmaceutics17070834 (PMC12298000; doi:10.3390/pharmaceutics17070834)
Supplement: Supplementary file 1 [file pharmaceutics-17-00834-s001.zip › pharmaceutics-3697766-supplementary.pdf]

Survey Study to Identify the Maximum Acceptable Numbers of 2 mm and 3 mm Mini-Tablets for Short-, Middle-, and Long-Term Treatments in Acutely and Chronically Sick Children of Different Age Groups Below 18 YearsManfred Wargenau[1], Eva Mutzke[2], Lucas-Sebastian Spitzhorn[1], Sibylle Reidemeister[3], Ingrid Klingmann[4], and Viviane Klingmann[2]*

Supplementary

Table S1. Descriptive statistics for number of mini-tablets (Cohorts 1 to 4).

Regimen	Cohort	No. of 2 mm MT<br>Mean (SD)<br>Median * (Range)	No. of 3 mm MT<br>Mean (SD)<br>Median * (Range)
SD	Acutely sick<br>Age: 6–<12 y	85.0 (± 57.8)<br>76.25 (11-200)	50.6 (± 35.3)<br>50.0 (1-150)
	Chronically sick<br>Age: 6-<12 y	120.6 (± 68.4)<br>125.0 (10-200)	80.5 (± 64.5)<br>72.5 (10-200)
	Acutely sick<br>Age: 12-<18 y	198.3 (± 91.9)<br>200.0 (70-300)	143.8 (± 96.9)<br>136.25 (35-300)
	Chronically sick<br>Age: 12–<18 y	242.1 (± 96.7)<br>250.0 (70-400)	152.3 (± 79.1)<br>150.0 (35-300)
1W	Acutely sick<br>Age: 6–<12 y	78.8 (± 57.1)<br>70.0 (11-200)	53.5 (± 43.2)<br>50.0 (1-200)
	Chronically sick<br>Age: 6–<12 y	117.1 (± 70.5)<br>112.5 (10-200)	80.1 (± 64.0)<br>72.5 (10-200)
	Acutely sick<br>Age: 12–<18 y	197.3 (± 91.1)<br>200.0 (50-300)	130.2 (± 79.1)<br>125.0 (35-300)
	Chronically sick<br>Age: 12–<18 y	230.2 (± 100.8)<br>225.0 (35-400)	146.0 (± 83.8)<br>145.0 (35-300)
12M	Acutely sick<br>Age: 6–<12 y	68.8 (± 58.2)<br>52.5 (11-200)	48.9 (± 48.4)<br>37.5 (0-200)
	Chronically sick<br>Age: 6–<12 y	117.8 (± 72.0)<br>115.0 (11-200)	80.5 (± 66.9)<br>73.75 (11-200)
	Acutely sick<br>Age: 12–<18 y	184.9 (± 83.5)<br>185.0 (62-300)	115.5 (± 65.8)<br>110.0 (35-300)
	Chronically sick<br>Age: 12–<18 y	212.7 (± 101.8)<br>201.25 (35-400)	126.3 (± 88.9)<br>117.5 (35-300)

SD = single administration, 1W = 3-times a day for one week, 12M = 3-times a day for 12 months, MT = mini-tablets, N = 24 for all groups, No. = number, SD = standard deviation, y = year* Median was calculated with Hodges-Lehman estimator (HL-median)

Table S2. Descriptive statistics for number of mini-tablets (Cohorts 5 - 14).

Regimen	Cohort	0-<2 y		2-<6 y		6-<12 y		12-<18 y	
		Mean number of mini-tablets (SD)<br>Median * (Range)							
		2 mm MT	3 mm MT	2 mm MT	3 mm MT	2 mm MT	3 mm MT	2 mm MT	3 mm MT
SD	Parents acutely sick children	17.7 (± 15.5)<br>15 (3-50)	11.7 (± 13.7)<br>8 (1-50)	51.3 (± 34.2)<br>50 (10-100)	28.3 (± 19.9)<br>29 (5-60)	97.9 (± 51.6)<br>87.5 (25-200)	46.8 (± 27.6)<br>50 (11-100)	225.8 (± 84.3)<br>225 (70-300)	140.6 (± 67.7)<br>140 (35-300)
	Parents chronically sick children	21.0 (± 16.9)<br>20 (3-50)	10.6 (± 10.6)<br>7.75 (1-35)	44.6 (± 32.0)<br>37.5 (5-100)	23.7 (± 25.9)<br>16.75 (5-100)	126.9 (± 65.4)<br>130 (25-200)	84.3 (± 60.1)<br>75 (11-200)	231.9 (± 96.4)<br>225 (35-400)	138.1 (± 94.7)<br>122.5 (35-300)
	Nurses	20.5 (± 13.4)<br>20 (3-50)	10.8 (± 10.6)<br>10 (0-50)	51.9 (± 28.9)<br>50 (15-100)	28.5 (± 23.3)<br>25 (5-100)	116.3 (± 59.1)<br>115 (25-200)	83.1 (± 61.1)<br>75 (15-200)	232.3 (± 88.0)<br>225 (50-300)	171.3 (± 88.3)<br>175 (35-300)
	Pediatricians	28.3 (± 14.6)<br>27.5 (3-50)	14.3 (± 9.0)<br>15 (0-35)	62.5 (± 29.0)<br>62.5 (5-100)	35.9 (± 19.1)<br>35 (5-75)	139.0 (± 62.8)<br>150 (11-250)	100.7 (± 57.7)<br>100 (11-200)	278.3 (± 120.1)<br>300 (25-500)	201.5 (± 98.4)<br>200 (20-400)
1W	Parents acutely sick children	17.5 (± 16.2)<br>15.75 (3-50)	7.9 (± 7.1)<br>6.5 (1-30)	49.8 (± 34.3)<br>50 (5-100)	26.8 (± 20.0)<br>27.5 (0-60)	90.0 (± 54.9)<br>87.5 (11-200)	43.7 (± 26.5)<br>40 (11-100)	199.2 (± 89.6)<br>200 (35-300)	114.8 (± 69.5)<br>110 (35-300)
	Parents chronically sick children	18.7 (± 16.7)<br>16.5 (3-50)	8.9 (± 9.5)<br>6.5 (1-35)	43.1 (± 32.1)<br>37.5 (5-100)	23.6 (± 25.8)<br>15.5 (3-100)	107.5 (± 68.5)<br>110 (20-200)	70.8 (± 57.1)<br>62.5 (11-200)	212.5 (± 97.3)<br>225 (35-400)	132.1 (± 90.3)<br>118.75 (35-300)
	Nurses	16.8 (± 10.1)<br>15.5 (3-40)	10.1 (± 8.9)<br>10 (0-40)	43.8 (± 30.8)<br>42.5 (5-100)	24.8 (± 20.5)<br>20 (5-75)	98.5 (± 57.0)<br>90 (25-200)	71.9 (± 52.5)<br>62.5 (15-200)	210.0 (± 87.8)<br>212.5 (50-300)	146.5 (± 79.3)<br>140 (35-300)
	Pediatricians	23.0 (± 12.6)<br>22.5 (3-50)	11.8 (± 8.6)<br>11.5 (0-35)	47.9 (± 26.0)<br>50 (5-100)	26.9 (± 16.0)<br>25 (5-75)	126.3 (± 60.9)<br>125 (20-250)	82.3 (± 48.4)<br>80 (15-200)	257.9 (± 113.0)<br>250 (20-500)	182.3 (± 94.7)<br>175 (15-400)
12M	Parents acutely sick children	15.6 (± 14.7)<br>15 (1-50)	8.2 (± 8.7)<br>6.25 (1-30)	45.7 (± 34.1)<br>47.5 (5-100)	23.0 (± 16.1)<br>22.5 (0-50)	91.3 (± 53.6)<br>87.5 (11-200)	41.6 (± 26.6)<br>37.5 (11-100)	182.5 (± 78.7)<br>175 (35-300)	103.5 (± 68.2)<br>92.5 (35-300)
	Parents chronically sick children	19.3 (± 18.9)<br>16.25 (2-70)	9.9 (± 11.7)<br>8 (1-50)	41.9 (± 33.8)<br>35 (5-100)	22.1 (± 26.3)<br>15 (3-100)	104.3 (± 73.6)<br>105.5 (11-200)	64.8 (± 54.7)<br>55.5 (11-200)	209.2 (± 104.1)<br>200 (35-400)	132.7 (± 93.8)<br>122.5 (35-300)
	Nurses	21.0 (± 16.1)<br>20 (2-50)	13.0 (± 12.8)<br>11 (0-50)	46.5 (± 35.9)<br>42.5 (5-120)	30.3 (± 28.4)<br>22.5 (5-100)	105.8 (± 66.0)<br>112.5 (25-200)	78.6 (± 65.6)<br>62.5 (11-200)	206.3 (± 86.3)<br>200 (60-300)	146.7 (± 80.7)<br>140 (35-300)
	Pediatricians	19.9 (± 11.9)<br>20 (3-50)	9.7 (± 7.9)<br>9 (0-35)	43.8 (± 27.0)<br>42.5 (10-100)	24.1 (± 15.4)<br>22.5 (3-75)	118.8 (± 63.9)<br>112.5 (10-250)	74.4 (± 49.3)<br>70 (5-200)	247.3 (± 117.9)<br>250 (15-500)	156.0 (± 92.9)<br>150 (5-400)

SD = single administration, 1W = 3-times a day for one week, 12M = 3-times a day for 12 months, MT = mini-tablets, SD = standard deviation, y = year, N = 24 for all groups, * Median was calculated with Hodges-Lehman estimator (HL-median)
